# The interplay of directional information provided by unpolarised and polarised light in the heading direction network of the diurnal dung beetle *Kheper lamarcki*

**DOI:** 10.1242/jeb.243734

**Published:** 2022-02-10

**Authors:** Lana Khaldy, James J. Foster, Ayse Yilmaz, Gregor Belušič, Yakir Gagnon, Claudia Tocco, Marcus J. Byrne, Marie Dacke

**Affiliations:** 1 Lund Vision Group, Department of Biology, Lund University, Sölvegatan 35, 223 62 Lund, Sweden; 2 Zoology II, Biozentrum, University of Würzburg, Am Hubland, 97074 Würzburg, Germany; 3 Department of Biology, Biotechnical Faculty, University of Ljubljana, SI-1000 Ljubljana, Slovenia; 4 School of Animal, Plant and Environmental Sciences, University of the Witswatersrand, 1 Jan Smuts Avenue, Braamfontein, Johannesburg 2000, South Africa

**Keywords:** Directional cues, Compass, Weighting, Heading direction network, Navigation, Degree of polarisation

## Abstract

The sun is the most prominent source of directional information in the heading direction network of the diurnal, ball-rolling dung beetle *Kheper lamarcki*. If this celestial body is occluded from the beetle's field of view, the distribution of the relative weight between the directional cues that remain shifts in favour of the celestial pattern of polarised light. In this study, we continue to explore the interplay of the sun and polarisation pattern as directional cues in the heading direction network of *K. lamarcki*. By systematically altering the intensity and degree of the two cues, we effectively change the relative reliability as they appear to the dung beetle. The response of the beetle to these modifications allows us to closely examine how the weighting relationship of these two sources of directional information is influenced and altered in the heading direction network of the beetle. We conclude that the process by which *K. lamarcki* relies on directional information is very likely done based on Bayesian reasoning, where directional information conveying the highest certainty at a particular moment is afforded the greatest weight.

## Abstract (precis)

**Summary:** The diurnal dung beetle *K. lamarcki* relies on directional information in a Bayesian manner, affording the greatest weight to directional information conveying the highest certainty at the given moment.

## INTRODUCTION

Combining information from several different sensory cues can reduce the effect of noise in a system, allowing for greater accuracy of the behavioural output (Cheng et al., 2007; Deneve and Pouget, 2004). Within the realm of navigation, multisensory integration provides a robust navigational toolkit that lowers directional uncertainty; rock ants follow less tortuous routes when landmarks are visible (Hunt et al., 2018) and desert ants are better at localising their nest when olfactory cues are present (Huber and Knaden, 2017). Depending on the context and conditions under which the animal finds its way, directional information from multiple sensory cues can often be integrated, operating in parallel (Buehlmann et al., 2020). In this way, navigational performance will not be compromised if directional information from one source is disrupted. At high solar elevations, when directional information from the sun is deemed unreliable (Dacke et al., 2014), dung beetles rely on directional information from the wind to guide their straight-line orientation across the savanna (Dacke et al., 2019). Similarly, *Myrmica* ants, that predominantly depend on directional information from visual cues when negotiating a maze, resort to olfactory cues for directional information as the light intensity decreases and visual information becomes less reliable (Cammaerts, 2012).

The process by which orienting and navigating insects integrate multiple sources of directional information is very likely done according to Bayes’ theorem (Körding, 2007; Körding and Wolpert, 2006): directional information conveying the highest certainty at any given moment is afforded the greatest weight in the navigational network of the animal. In homing ants, which find their way back to their nest by path integration (PI) and landmark guidance (LG), the weighting relationship of the PI and LG will shift in favour of the former as the ants are displaced further from their nest (Wystrach et al., 2015). With growing distance, the surrounding visual scenery becomes increasingly unfamiliar, while at the same time the ant's PI vector becomes longer, providing a stronger, more reliable source of information. Along the same line of reasoning, if two directional cues of equal weight are set in conflict, this should result in an intermediate direction between the two sources of information. This outcome is also observed in homing ants when the apparent e-vector direction of the celestial pattern of polarised light is set in conflict with the artificial panorama (Freas et al., 2017; Reid et al., 2011) or the artificial panorama is set in conflict with other celestial cues (Legge et al., 2014; Wystrach et al., 2015).

For the dung beetle *Kheper lamarcki*, the sun is naturally the most prominent directional compass cue in its heading direction network (Dacke et al., 2013a; 2014; el Jundi et al., 2015; Khaldy et al., 2019a; 2019b; Smolka et al., 2016)*.* If the position of the sun is experimentally set in conflict with other celestial cues (with the aid of a mirror), *K. lamarcki* changes its bearing by 180 deg in response to this positional change (Dacke et al., 2014). Comparably, if the view of the sun is blocked (by a shading board), and the e-vector direction of the celestial polarised light is turned by 90 deg with a polariser, this beetle turns in accordance with the 90 deg positional change of the e-vector. Thus, when the sun is out of sight, the relative weight between the remaining directional cues shifts in favour of the celestial pattern of polarised light (el Jundi et al., 2014).

In this study, we explored the interplay of the sun and the polarisation pattern as directional cues in the heading direction network of the beetle. We also set out to measure the spectral sensitivity of the DRA of *K. lamarcki*, following the unusual finding of UV and green receptors in the dorsal rim area (DRA) of a closely related diurnal dung beetle, *Pachysoma striatum* (Dacke et al. 2002). By altering the intensity and degree of the presented cues, we effectively change their reliability as they appear to the dung beetle, allowing us to examine how the weighting relationship of these two sources is influenced and altered by their reliability in the heading direction network of the beetle.

## MATERIALS AND METHODS

### Collection and maintenance of animals

Beetles of the diurnal species *Kheper lamarcki* (MacLeay 1821) were collected using dung-baited pit-fall traps at Stonehenge game farm (26°23'56″S, 24°19'36″S), South Africa, in November 2020 and February 2021. Once collected, beetles were transported to the Department of Biology, Lund University, Sweden, and housed in large plastic bins (50×36×27 cm) in a light- and temperature-controlled room, under a 12 h:12 h light:dark cycle, at a room temperature of 26°C and fed with fresh dung every third day.

### Statistics

Circular data are reported as means±1 circular s.d. Circular statistics on measured data were performed using Oriana 4.0 (Kovach Computing Services, Anglesey, UK). The distribution of exit angles was analysed using Rayleigh's uniformity test for circular data (Batschelet, 1981). Changes in direction between treatments were calculated by measuring the absolute mean angular difference of the five exits preceding and the five exits following the treatment. This applied for all treatments apart from the condition where a dim ersatz sun was presented in combination with a 64% polarised overhead light. Here, the distribution of exit angles was analysed by calculating the mean vector length (*r*) of the first five consecutive rolls. In conditions where the animal displayed bimodal distribution of exit angles, angles were projected back onto the semi-circle surrounding the direction of most exit angles. A Mann–Whitney rank-sum test was used to determine whether the absolute angular difference between a treatment was significantly higher in the test condition (position of stimulus is changed by 90 deg between treatments) compared with the control condition (position of stimulus remains unchanged between treatments). The Mann–Whitney test was thus used to test whether the animal turned with the stimulus. To test for homogeneity on two or more samples, a Mardia–Watson–Wheeler test was used. Generalised linear model (http://www.rstudio.com/) was used to assess the relationship between degree of polarisation and probability of a turn (>45 deg).

### Physiology

In preparation for intracellular recordings from the photoreceptors of dark-adapted individuals, the beetles were immobilised with beeswax and resin at room temperature (for details, see Belušič et al., 2017) and mounted on a goniometric *XYZ*-stage that carried a micromanipulator (Sensapex, Oulu, Finland). A 50 μm diameter Ag/AgCl wire (inserted into the head capsule next to the eye) served as a reference electrode. Microelectrodes (Sutter, Novato, CA, USA) filled with 3 mol l^−1^ KCl (resistance 100–150 MΩ) were inserted into the eye via a small triangular hole in the cornea, ventral of the (expected) DRA. The signal was amplified using an SEC 10 LX amplifier (Npi electronic, Tamm, Germany) and a Cyber Amp 320 (Axon Instruments, Union City, CA, USA) and finally digitised via a Micro 1401 (CED, Cambridge, UK). Spectral stimulation was provided with an LED array (‘LED synth’; Belušič et al., 2017), and with light from a xenon arc lamp (XBO, Cairn Research Ltd, Faversham, UK) filtered with a monochromator (B&M, Limburg, Germany). The light sources were tuned to emit equal numbers of photons at every wavelength (‘isoquantal’ mode). A UV transmissive polarisation filter (OUV2500, Knight Optical, Harrietsham, UK) was mounted in a motorised rotator (Qioptiq, Göttingen, Germany) and inserted into the stimulation beam to facilitate measurement of polarisation sensitivity. All cells were first quickly stimulated with the LED synth, to determine their spectral sensitivity within 2 s, after which their polarisation sensitivity was measured at their sensitivity peak (360 or 500 nm). This was followed by measuring the intensity–response function and a detailed spectral scan with a monochromator. The response amplitude of single cells was transformed to sensitivity by means of an intensity–response function and a reverse Hill transformation (Belušič et al., 2017). Polarisation sensitivity (PS) was calculated as the ratio between the sensitivity maximum and minimum, i.e. PS=*S*_max_/*S*_min_ (Bernard and Wehner, 1977). Some cells were lost during the spectral scan, hence the number of cells (*N*) with measured polarisation sensitivity is higher than *N* cells with measured spectral sensitivity.

### Light measurements

**Fig. 1.**
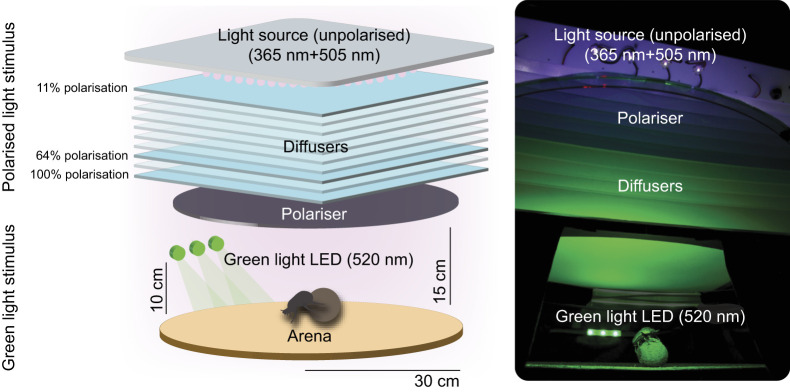
**Experimental setup.** Schematic depiction (left) and image (right) of the experimental setup with an overhead polarised light stimulus and a laterally presented green light stimulus. The overhead light stimulus consisted of an unpolarised light fixture (the light source) of 80 UV light-emitting diodes (365 nm) and 21 cyan light-emitting diodes (510 nm) centred on a square-shaped aluminium plate, along with 10 sheets of diffusers (Plexiglas^®^), 1 cm apart, and a polarisation filter (‘polariser’). The degree of polarisation produced by the overhead light varied depending on the placement of the polarisation filter within the stack of diffusers. The polariser could be placed in three different positions within the setup (highlighted in blue in the figure): (i) above the 10 sheets of Plexiglas (11% polarisation), (ii) before the 8th sheet (64% polarisation), or (iii) after the 10th sheet (100% polarisation). The overhead light stimulus was suspended 15 cm above a circular arena of 60 cm diameter. The green unpolarised light stimulus (520 nm) consisted of three horizontally aligned LEDs (9.5 cm×0.5 cm) presented to the beetle from either of the four sides of the arena (0, 90, 180 and 270 deg), 30 cm from the arena centre, at a height of 10 cm.

Irradiance was measured by placing a cosine corrector coupled to a spectrometer via a calibrated light guide (cosine corrector: CC-3-UV-T; spectrometer: QE65000; light guide: P600-2-UV-VIS, Ocean Optics Inc., Dunedin, FL, USA) in the centre of the arena, 8 cm above the arena floor (corresponding to the position of the beetle on top of its dung ball) (Fig. 1). Degree of polarisation of the light was analysed by a UV-transmissive linear polariser (Glan-Thompson; GTH5M-A: Thorlabs GmbH, Dachau, Germany) coupled to a spectrometer via a light guide (spectrometer: FLAME-S-UV-VIS; light guide: P1000-2-UV-VIS; Ocean Optics). To avoid measuring off-axis light, the beam of light was sampled through an opaque lens tube (Foster et al., 2018).

### Experimental setup

The experimental setup consisted of (i) an overhead polarised light stimulus, raised 15 cm above a flat, circular, sand-painted 60 cm diameter arena, and (ii) a green light stimulus presented from the side, 30 cm from the arena centre, at a height of 10 cm (Fig. 1).

#### Polarised light stimulus

Having identified UV and green receptors with high polarisation sensitivity in the dorsal region of the dorsal eye of *K. lamarcki*, we decided to stimulate the DRA with a combination of UV and cyan light. Eighty UV light-emitting diodes (LZ1-10UV00-0100; emission peak 365 nm, LedEngin Inc., San Jose, CA, USA) and 21 cyan light-emitting diodes (LXML-PE01-0070; emission peak 505 nm, Lumileds, San Jose, CA, USA) were mounted and arranged in a circular pattern (58 cm diameter) centred on a square-shaped aluminium plate (60×60×0.2 cm), resting on a custom-built shelf mounted 50 cm above the arena floor. Ten sheets of Plexiglas^®^ (60×60×0.3 cm, Plexiglas^®^ Solar 2458, EBLA-GmbH, Appenweier, Germany), arranged in a stacked fashion, 1 cm apart, were placed 7.5 cm below the UV/cyan light fixture. Each sheet of Plexiglas was sand blasted on one side (facing downward) to act as a diffuser (Egri et al., 2016). A circular, UV-transmissive polarisation filter (BVO UV Polarizer, Bolder Vision Optik^©^, Boulder, CO, USA; 60 cm diameter) was placed at three different positions within the setup: (i) above the 10 sheets of Plexiglas (11% polarisation), (ii) above the 8th sheet of Plexiglas (64% polarisation), or (iii) below the 10th sheet of Plexiglas (100% polarisation) (Fig. 1). As a result of the experimental design, the animal was no less than 7–12 cm away from the overhead stimulus (see Fig. 1). Thus, the overhead stimulus subtended a visual angle of approximately 136–154 deg from the arena centre throughout all conditions. The combined polarised light stimulus had an irradiance of 1.26×10^15^ photons cm^−2^ s^−1^: cyan alone 2.39×10^14^ photons cm^−2^ s^−1^ and UV alone 1.04×10^15^ photons cm^−2^ s^−1^. This applied to all conditions where the polarised light stimulus was used, except for the condition in which the intensity of the polarised light stimulus was lowered. In this condition, the irradiance for 365 nm was lowered to 3.18×10^13^ photons cm^−2^ s^−1^ while that for 505 nm remained unchanged.

#### Green light stimulus

The beetles were also presented with a green unpolarised light source (a previously documented replacement for the sun in the heading direction network of the beetle (el Jundi et al., 2015) consisting of three horizontally aligned LEDs (Adafruit DotStar Digital LED Strip; emission peak 520 nm, Adafruit Industries, New York, NY, USA). This ersatz sun (9.5 cm×0.5 cm) was presented to the beetle from either of the four sides of the arena (0, 90, 180 and 270 deg) (Fig. 1) at an intensity of 1.72×10^13^ or 1.02×10^12^ photons cm^−2^ s^−1^.

When evaluating the isolated response to the ersatz sun, the polariser was removed from the overhead light stimulus, resulting in an unpolarised overhead stimulus with the same spectrum. This applied to all conditions except for the condition where the response to the dim ersatz sun was evaluated in which no overhead light was presented.

### Experimental method

A beetle was placed alongside its dung ball, in the centre of the circular arena, and allowed to roll its ball to the perimeter where the exit bearing was noted. The beetle was then removed from its ball and placed back in the centre of the arena alongside its ball. This procedure was repeated 5 times. Beetles not successful in adhering to their bearing over their initial five exits (*P*<0.1, Rayleigh uniformity test) were excluded from any further experiments (for an overview of the behavioral outcome in each paradigm, see Table S1).

### Manipulation of directional input

#### Polarised light

Once the beetle had exited the arena 5 times, the polarisation filter was either kept in place (control) or turned by 90 deg (test) before the beetle was allowed to exit the arena 5 additional times. The initial orientation of the filter alternated for each beetle, with every second beetle starting with the polarisation filter aligned to the 0–180 deg direction of the circular arena, and every other beetle with the filter aligned perpendicular to this.

#### Ersatz sun

The initial position of the ersatz sun was placed in one of four positions around the arena (0, 90, 180 or 270 deg). Once the beetle had exited the arena 5 times with the ersatz sun in a fixed position, the apparent position of this light was either held stationary or changed by 90 deg, in relation to its previous position, before the beetle was allowed to exit an additional 5 times.

## RESULTS

### Ball-rolling dung beetles can orient to a green light stimulus

**Fig. 2.**
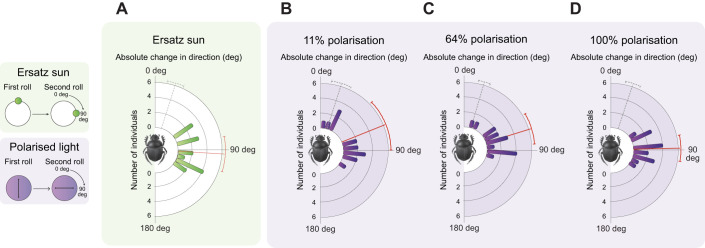
**Response to directional change of compass cues in *Kheper lamarcki*.** The dung beetle was allowed to roll its dung ball from the centre of a 60 cm diameter arena (A) in the presence of a lateral green light source (ersatz sun) in combination with an overhead unpolarised light source, or (B–D) in the presence of a single overhead polarised light source (B: 11% polarisation; C: 64% polarisation; D: 100% polarisation). Once the beetle had reached the periphery of the arena, it was removed from its dung ball and placed back in the centre alongside its ball. This procedure was repeated 5 times. After the fifth exit from the arena, the apparent position of the ersatz sun (A) or the e-vector direction of the artificial band of the overhead polarised light source (B–D) was turned by 90 deg (test), or remained in position (control). The beetle was then allowed to exit the arena 5 more times. The absolute angular change between the mean direction of the five exits prior to the treatment and the mean direction of the five exits following the treatment in the test condition is depicted as coloured bars in all graphs. Under all four conditions, dung beetles changed bearing direction in accordance with the 90 deg angular change of the stimulus presented (red vector, all graphs). The absolute angular difference between the mean direction of the five exits prior to the treatment and the five exits following the treatment during the control condition is represented by a grey dotted vector in each graph. Error bars represent one circular standard deviation.

When the position of the ersatz sun was changed by 90 deg between two trials (test), the beetles changed their headings accordingly (mean±s.d. 93.55±25.97 deg, *N*=15; Fig. 2A), with a significantly larger turning angle compared with the control condition when the ersatz sun remained stationary (14.76±9.77 deg, *N*=15; Fig. 2A, grey dotted line) (Mann–Whitney rank sum test, *W*=345, *P*<0.001, *z*=4.65, *N*=15). This clearly demonstrates that *K. lamarcki* can steer with reference to the green light source provided in the experimental arena.

However, when the ersatz sun was lowered 10-fold in intensity (from 1.72×10^13^ to 1.02×10^12^ photons cm^−2^ s^−1^), the beetles failed to orient to the ersatz sun in the presence of an overhead unpolarised light (Fig. 4H). Only when no overhead light was present did the beetle show a response to the turned stimulus (Fig. 4G). This indicates that when the overhead light is present, the light information provided by the dim ersatz sun cannot be distinguished from the background light, and the beetle fails to orient.

### Ball-rolling dung beetles orient with the same precision under a wide range of degrees of overhead polarisation

**Fig. 3.**
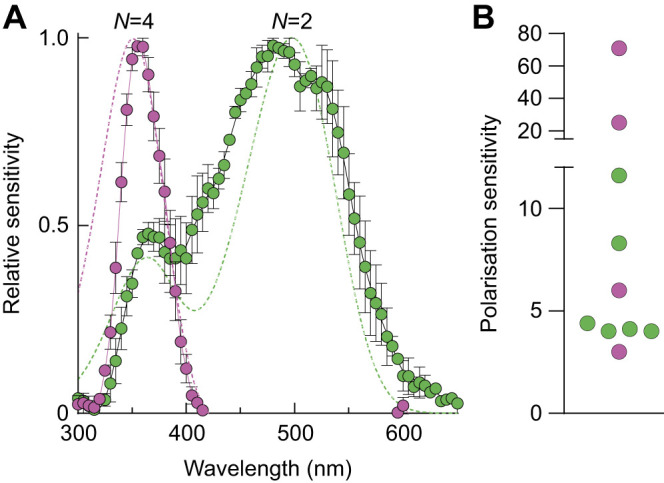
**Intracellular photoreceptor recordings in the dorsal region of the dorsal eye of *K. lamarcki.*** Intracellular recordings in the dorsal region of the dorsal eye revealed two types of spectrally distinct, but highly polarisation-sensitive photoreceptors: UV and green sensitive. (A) Spectral sensitivity of the UV-sensitive photoreceptors (λ_max_≈350 nm; pink) and green-sensitive photoreceptors (λ_max_≈500 nm; green), fitted with rhodopsin nomograms (pink dashed line: λ_max_=352 nm; green dashed line: λ_max_=501 nm). (B) Polarisation sensitivity of UV-sensitive (pink) and green-sensitive (green) photoreceptors.

Intracellular photoreceptor recordings in the dorsal region of the dorsal eye of *K. lamarcki* revealed two types of spectrally distinct, but highly polarisation-sensitive photoreceptors: one sensitive in the ultraviolet (UV, λ_max_≈350 nm) and one in the green (λ_max_≈500 nm) range of the electromagnetic spectrum (Fig. 3A). Both photoreceptor types had high or very high polarisation sensitivities (PS_UV_=3, 6, 25, 71, mean±s.d. 26.3±31.4; PS_G_=4, 4, 11.6, 8.3, 4.4, 4.12, mean±s.d. 6.1±3.2) (Fig. 3B).

When the artificial, overhead band of polarised light (365 and 505 nm) was turned by 90 deg, the beetles turned in accordance with this under all three levels of polarisation presented (11% polarisation: 67.96±38.90 deg; 64% polarisation: 72.80±23.33 deg; 100% polarisation: 88.74±19.35 deg; *N*=15) (Fig. 2B–D). This turning angle differed significantly from when beetles were instead presented with the artificial band of polarisation in the same position for two consecutive trials (control) (11%: 17.79±14.87 deg; 64%: 16.56±8.73 deg; 100%: 16.57±10.01 deg) (Fig. 2B–D, grey dotted line) (Mann–Whitney rank sum test, 11%: *W*=307, *P*=0.002, *z*=3.07; 64%*: W*=340, *P*<0.001, *z*=4.43; 100%: *W*=345, *P*<0.001, *z*=4.65, *N*=15). Although no significant difference in response could be found between the three conditions for either the test or control conditions (control: *P*=0.17; test: *P*=0.69, Mardia–Watson–Wheeler test, *N*=15), the data show a significant correlation between the degree of polarisation and the probability of a turn (>45 deg), demonstrating that turning probability increases with increasing degree of polarisation (GLM, *z*=2.23, AIC=36.969, *P*=0.0257) (Fig. S1).

### The weighting relationship between the ersatz sun and polarised light is highly dynamic

**Fig. 4.**
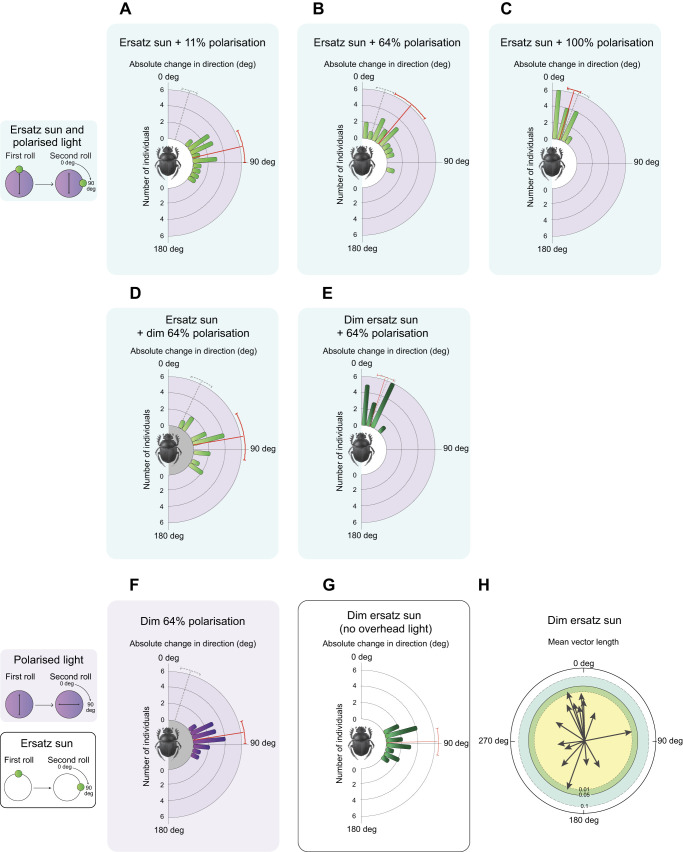
**Response of *K. lamarcki* to a directional change of the ersatz sun in the presence of polarised light.** The dung beetle was allowed to roll its dung ball from the centre of a 60 cm diameter arena in the presence of a laterally presented green light source (ersatz sun) in combination with an overhead polarised light. (A) Ersatz sun in the presence of 11% polarisation; (B) ersatz sun in the presence of 64% polarisation; (C) ersatz sun in the presence of 100% polarisation; (D) ersatz sun in the presence of 64% polarisation of lower UV light intensity; (E) lower intensity ersatz sun in the presence of 64% polarisation; (F) 64% polarisation of lower UV light intensity; (G) lower intensity ersatz sun in the absence of overhead unpolarised light; (H) lower intensity ersatz sun in the presence of overhead unpolarised light. Once the beetle had reached the periphery of the arena, it was removed from its dung ball and placed back in the centre alongside its ball. This procedure was repeated 5 times. After the fifth exit from the arena, the apparent position of the ersatz sun (A–E, G) or the e-vector direction of the artificial band of the overhead polarised light source (F) was turned by 90 deg (test), or remained in position (control), or the treatment was finished (H). The beetle was then allowed to exit the arena 5 more times. The absolute angular change between the mean direction of the five exits prior to the treatment and the mean direction of the five exits following the treatment during the test condition is depicted as coloured bars in A–G. When exiting in the presence of an ersatz sun under 11% polarised light, *K. lamarcki* changed its bearing in accordance with the 90 deg angular turn of the ersatz sun (A). In contrast, when exiting in the presence of an ersatz sun under 100% polarised light, *K. lamarcki* did not respond to the positional change of the ersatz sun (C). If instead it was presented with an ersatz sun in the presence of 64% polarised light, the beetle showed an intermediate response to the azimuthal change of the stimulus (B). However, when the intensity of the 64% polarised light decreased 100-fold, the beetle again turned in response to the 90 deg turn of the ersatz sun (D). Comparably, when instead the intensity of the ersatz sun was decreased 10-fold and presented in combination with the full intensity of 64% overhead polarised light, the beetle did not respond to the turn of the ersatz sun (E). The absolute mean angular difference between the five exits prior to the treatment and the five exits following the treatment during the control condition is represented by a grey dotted vector in A–F. Error bars represent one circular standard deviation. The directedness of each individual in H is represented by the mean vector length (*r*) and is depicted by black arrows. The shorter the mean vector length, the less oriented the individual. The edges of the coloured circles in H indicate the required *r*-value for statistical significance: yellow: *P*<0.01, green: *P*<0.05, and blue: *P*<0.1.

To investigate the weighting relationship of directional information from the sun (here represented by an ersatz sun) and the directional information from polarised skylight (here represented as an overhead polarised light source), the beetles were presented with the two cues at the same time. When the ersatz sun was changed by 90 deg between trials (test), the beetles only turned in accordance with this change when the degree of the polarised light presented from above (that remained in place) was set to its lowest setting of 11% polarisation (control: 16.32±10.46 deg; test: 77.13±21.94 deg, Mann–Whitney rank sum test, *W*=345, *P*<0.001, *z*=4.64, *N*=15) (Fig. 4A). When rolling under the highest degree of overhead polarised light (100% polarisation), the beetles maintained their original bearing, seemingly ignoring the 90 deg change of the azimuthal position of the ersatz sun (control: 16.55±8.66 deg; test: 16.72±9.25 deg, Mann–Whitney rank sum test, *W*=231, *P*=0.97, *z*=−0.042, *N*=15) (Fig. 4C). *Kheper lamarcki* thus steered in reference to the ersatz sun when it was presented together with a low degree of overhead polarisation and in reference to the e-vector direction of the polarised light when it was presented together with a high degree of overhead polarisation. In the presence of a polarised light stimulus of 64% polarisation, the beetles again changed their bearings, but now to a lesser degree (40.01±26.06 deg, *N*=15) (Fig. 4B). Together, these results suggest that the weighting relationship between directional information from the ersatz sun and the polarised light source changes with a change in the degree of polarised light.

### The light intensity of the directional cues influences their weighting relationship

Given that the beetles neither conclusively maintained their original bearing nor turned in accordance with the 90 deg azimuthal change of the ersatz sun when the overhead light was 64% polarised, we next lowered the intensity of the polarised UV light approximately 100-fold (from 1.04×10^15^ to 3.18×10^13^ photons cm^−2^ s^−1^). This allowed us to investigate whether the intensity of the polarised light would also influence the weighting relationship between the two sources of directional information.

To confirm that the beetles were still able to respond to the e-vector rotation of this dimmer stimulus, we first presented the overhead light cue in isolation, either stationary (control; 17.57±13.64 deg, *N*=15) or with a 90 deg rotation between trials (81.21±14.98 deg, *N*=15) (Fig. 4F). The beetles still turned in accordance with the turn of the polarisation axis of the overhead light (Mann–Whitney rank sum test, *W*=345, *P*<0.001, *z*=4.65, *N*=15). We further found that there was no significant difference in orientation performance between the groups of beetles orienting under the high and low intensity of the polarised light stimulus. This held true for both the control and the test conditions (control: *P*=0.22, *W*=3.02; test: *P*=0.39, *W*=1.87, Mardia–Watson–Wheeler test, *N*=15).

When presented with the ersatz sun in combination with the lower intensity overhead polarised light, the beetles now turned in accordance with the positional change of the ersatz sun (control: 16.78±14.58 deg; test: 80.20±32.22, *N*=15) (Fig. 4D) (Mann–Whitney rank sum test, *W*=340, *P*<0.001, *z*=4.44, *N*=15). This response was significantly different to the observed response when beetles were presented with an ersatz sun in combination with the full intensity polarisation stimulus of 64% (*P*=0.026, *W*=7.28, Mardia–Watson–Wheeler test, *N*=15). This indicates that when the intensity of the polarised light source was lowered, the weighting relationship between the two sources of information shifted towards directional information from the ersatz sun.

Correspondingly, when instead the intensity of the ersatz sun was reduced and presented in combination with the full intensity polarisation stimulus of 64%, the beetles were unresponsive to the positional change of the dim ersatz sun (see Fig. 4H). Instead, the beetles adhered to their original direction (Fig. 4E), similar to when presented a full intensity ersatz sun in combination with 100% overhead polarised light (Mann–Whitney rank sum test, *W*=191, *P*<0.59, *z*=−0.52, *N*=15) (cf. Fig. 4C). Thus, when the intensity of the overhead polarised light or the ersatz sun was lowered, the weighting relationship between the two sources shifted away from the directional information provided by the dimmed cue.

## DISCUSSION

### Evidence of UV and green polarisation-sensitive photoreceptors in the DRA of *K. lamarcki*

Under a clear, sun-lit sky, the celestial polarised light pattern is highly distinguishable across all wavelengths of light. Under overcast skies or a tree canopy, the detection of this celestial pattern is most advantageous in the UV range (Barta and Horváth, 2004; Hegedüs et al., 2007a; Seliger et al., 1994; Wang et al., 2014). Perhaps it is because of this stability that most insects, including honeybees (Labhart, 1980), ants (Duelli and Wehner, 1973), earth-boring beetles (Frantsevich et al., 1977), butterflies (Stalleicken et al., 2006) and flies (Hardie et al., 1979) analyse this pattern through UV-sensitive photoreceptors. The unusually high polarisation sensitivity of 71 presented here for *K. lamarcki* in the UV (Fig. 3) is very likely a result of electrical inhibition in the photoreceptor cell (Weir et al., 2016) or possibly due to mutual filtering in the fused rhabdom between orthogonally oriented rhabdomeres (Heras and Laughlin, 2017).

Interestingly, for the diurnal dung beetle *K. lamarcki*, our findings show evidence for polarisation-sensitive photoreceptors in UV as well as green-sensitive cells (Fig. 3). Furthermore, the rare finding of two spectrally distinct, highly polarisation-sensitive photoreceptor classes (UV and green) for polarisation detection has also been suggested in the closely related, homing dung beetle, *Pachysoma striatum* (Dacke et al., 2002). *Kheper lamarcki* (as well as *P. striatum*) are active in open, dry habitats (Scholtz and Ranwashe, 2020), where the sky is clear and the degree of polarisation is high (Brines and Gould, 1982; Horváth et al., 2014). Under such conditions, the addition of green polarisation-sensitive cells could perhaps increase the overall polarisation sensitivity of the animal's eyes, much as has been suggested in nocturnal insects (Belušič et al., 2017; Eggers and Gewecke, 1993; Labhart et al., 1992). However, for now, we can only speculate on this matter.

### Response to the polarised light cue information as a function of its degree

When exiting the arena in the presence of an overhead polarised light source, presented in isolation, *K. lamarcki* showed a clear response to the 90 deg rotation of the artificial band of polarised light under 11%, 64% and 100% polarisation (Fig. 2B–D). In addition, the probability of a turning response (number of individuals that turn by 45 deg or more) decreased with a decreasing degree of polarisation (raw turn probability: 100% polarisation=15/15, 64% polarisation=13/15, 11% polarisation=10/15; Fig. S1), demonstrating a strong correlation between the degree of polarisation and turning response. The degree of polarised light is determined by the intensity of the electric field component in proportion to the light beam's overall intensity (Strutt, 1871; Suhai and Horváth, 2004) and can therefore act as a measure of signal strength: the higher the degree of polarisation, the stronger the signal. In crickets, the polarotactic response diminishes as the animal is presented with a stimulus of a lower degree of polarisation (Henze and Labhart, 2007; Labhart, 1996). If the response to polarisation is limited by receptor noise (Labhart, 1996), then a greater signal strength would lead to more polarisation-sensitive neurons being stimulated; thus, a high degree of polarised light is likely to generate a stronger output signal and further affect the weighting strategy of the beetle's heading direction network. This can also be observed in nature; during overcast conditions, when the degree of polarisation is severely diminished (Barta and Horváth, 2004; Horváth et al., 2014), the ability to maintain a straight rolling bearing is disrupted in diurnal and nocturnal dung beetles alike (Dacke et al., 2013a; 2013b). A similar correlation is also found in the nocturnal ball-rolling dung beetle *Scarabaeus satyrus* (Foster et al., 2019); when allowed to roll underneath an overhead polarised light source (similar to the polarised light source presented in this paper; Fig. 1) of differing degrees of polarised light, the ability of the beetle to maintain its exit bearing over consecutive rolls (orientation precision) lowered in correspondence with the degree of overhead polarised light presented.

### The intensity of the directional cue affects its reliability as a directional cue

In this study, we found that *K. lamarcki* can reliably extract and utilise directional information from polarised light of a degree as low as 11% (Fig. 2B), corresponding to the threshold limit suggested for its nocturnal cousin, *S. satyrus* (Foster et al., 2019). If coerced to roll on a moon-lit night, with the apparent position of the real moon covered from the beetle's field of view, the diurnal *K. lamarcki* does however fail to maintain a straight bearing (Smolka et al., 2016). It is important to note that the light intensity presented to the diurnal beetle in this study is three to four orders of magnitude higher than that presented to *S. satyrus* in the study by Foster et al. (2019), and nearly six orders of magnitude higher than the intensity of polarised light in the night sky (Foster et al., 2019; Johnsen et al., 2006). Insects that carry an ‘e-vector map’ (a neural map of the e-vector distribution across the sky relative to the position of the sun) could, at least in theory, rely solely on the direction of the e-vector of the polarised light for directional information (Brines and Gould, 1979; 1982; Labhart, 1988; 1996; Rossel and Wehner, 1984). Only when the noise of the visual signal outcompetes the difference between the orthogonally arranged groups of microvillar rhabdomeres does the intensity of the polarisation cue become an important factor (el Jundi and Homberg, 2012). Thus, the inability of *K. lamarcki* to steer straight according to the polarisation pattern surrounding the moon is very likely a result of the limitations of the animal's own sensory ecology; the eyes of *K. lamarcki* might just not be able to detect the polarised skylight pattern across the night sky.

Along similar lines, the integration of directional information from a point-light source is highly dependent on its intensity. When the position of the ersatz sun was changed by 90 deg, the beetles changed their headings accordingly (Fig. 2A). This was expected, as this outcome for *K. lamarcki* has been shown in several previous studies (Khaldy et al., 2019a; el Jundi et al., 2015; Smolka et al., 2016). However, if the same paradigm was presented to the beetle, but now with an ersatz sun of 10-fold lower intensity, the beetles could not maintain a straight bearing. Only when no overhead light was present would the beetles respond to the turned stimulus (Fig. 4G). This outcome suggests that when an overhead unpolarised light is present, the light information provided by the dim ersatz sun cannot be distinguished from the background. In this scenario, no directional information can be provided by the surroundings, and the beetles fail to orient. In contrast, when the same dim ersatz sun is presented in an otherwise darkened setup, the visual contrast between this light cue and the background is greater, thus providing enough visual directional information for orientation.

### Varying the reliability of the presented cue influences the relative weighting relationship

When presented with an ersatz sun in combination with an overhead polarised light source at 11% polarisation, all beetles turned in response to the azimuthal displacement of the ersatz sun (Fig. 4A). However, when the polarised light cue was presented in isolation, *K. lamarcki* was fully able to extract directional information from the weakly polarised light (Fig. 2B). We interpret this relative weighting of directional information, now in favour for the ersatz sun, as if this single bright light generates a stronger and more reliable directional signal relative to the artificial band of polarised light. This weighting relationship is directly comparable to that observed outdoors; when the apparent position of the sun is changed by 180 deg with the aid of a mirror, while simultaneously blocking the real sun from view under a natural sky, *K. lamarcki* will turn in response to the mirrored sun (Dacke et al., 2014; Khaldy et al., 2019a; 2019b). This means that the directional information from the sun dominates in its heading direction network, not only over the celestial polarisation information but also over all remaining skylight cues. However, with the apparent position of the sun shaded from view, which can occur naturally by cloud cover or experimentally using a shading board, these beetles instead follow the polarised light of the diurnal sky (el Jundi et al., 2014). Now, the distribution of the relative weight between the directional cues that remain shifts in favour of the polarised light input.

When instead presented with a fully (100%) polarised light source, in addition to the same laterally presented ersatz sun as above, the beetles no longer turned in response to a 90 deg azimuthal change of the ersatz sun. Their consistent orientation along the same bearing was now instead guided by the stable e-vector direction of the overhead polarised light (Fig. 4C). In this paradigm, directional information from the ersatz sun no longer dominates the heading direction network of the beetle, and the relative weighting between the two cues presented has shifted towards directional information from the polarised light cue. Our result clearly demonstrates that this species alters its weighting of cues in a context-dependent manner.

Because polarised skylight does not exceed 80% in the natural sky (Brines and Gould, 1982; Hegedüs et al., 2007b; Foster et al., 2019), the beetle will never be exposed to a fully polarised sky in nature. However, natural factors, such as clouds or vegetation, can reduce the reliability of the sun as a source of directional information, effectively shifting the weight attributed to this directional cue in the heading direction network of the beetle, similar to our 100% polarised light condition.

When next presented with 64% polarised overhead light in combination with the ersatz sun, *K. lamarcki* instead changed their bearings by about 45 deg in response to the 90 deg rotation of the light (Fig. 4B). Such an intermediate response, when two directional cues are set in conflict, can also observed in ants (Lebhardt and Ronacher, 2014; Legge et al., 2014). With light polarised to 64%, it consequently appears as if the two sources of input signal are providing directional information of similar reliability. Under specific conditions, this paradigm could also appear in nature. While dung beetles do not appear to interpret polarised skylight as necessarily perpendicular to the sun's azimuth, as bees and ants do (Rossel and Wehner, 1984; Fent, 1986), they still record a snapshot of available cues prior to rolling (el Jundi et al., 2016). If this snapshot includes horizontally polarised light from the opposite half of the sky, and cloud movement then obscures that region and reveals vertically polarized light closer to the sun, then the resulting conflict between polarised light and sunlight at the appropriate intensity range might reproduce the split in rolling behaviour that we observed here. However, as ball-rolling behaviour is undertaken over a short period of time (6 min; Dacke et al., 2019), we expect that this scenario would rarely occur.

When the intensity of the overhead polarised UV light is lowered, the beetles again turn with the ersatz sun (Fig. 4D). Interestingly, if presented with a dim ersatz sun in combination with the full intensity of 64% polarised overhead light, the beetles seemingly ignore the positional change of the dim ersatz sun and instead orient primarily according to the directional information from the overhead polarised light. Weakening the relative input of directional information from one cue thus effectively shifts the relative directional weighting between these two sources of information.

From the behavioural outcomes of our experiments, we can safely conclude that *K. lamarcki* integrates multiple sources of directional information in a Bayesian manner (Cheng et al., 2007; Körding, 2007; Körding and Wolpert, 2006), demonstrating that directional information conveying the highest certainty at any given moment is afforded the greatest weight in the navigational network of the animal.

## Supplementary Material

Supplementary information
