# Supplementary material for: The interplay of directional information provided by unpolarised and polarised light in the heading direction network of the diurnal dung beetle Kheper lamarcki
Source: J Exp Biol. 2022 Feb 10;225(3):jeb243734. doi: 10.1242/jeb.243734 (PMC8918814; doi:10.1242/jeb.243734)
Supplement: Supplementary information [file jexbio-225-243734-s1.pdf]

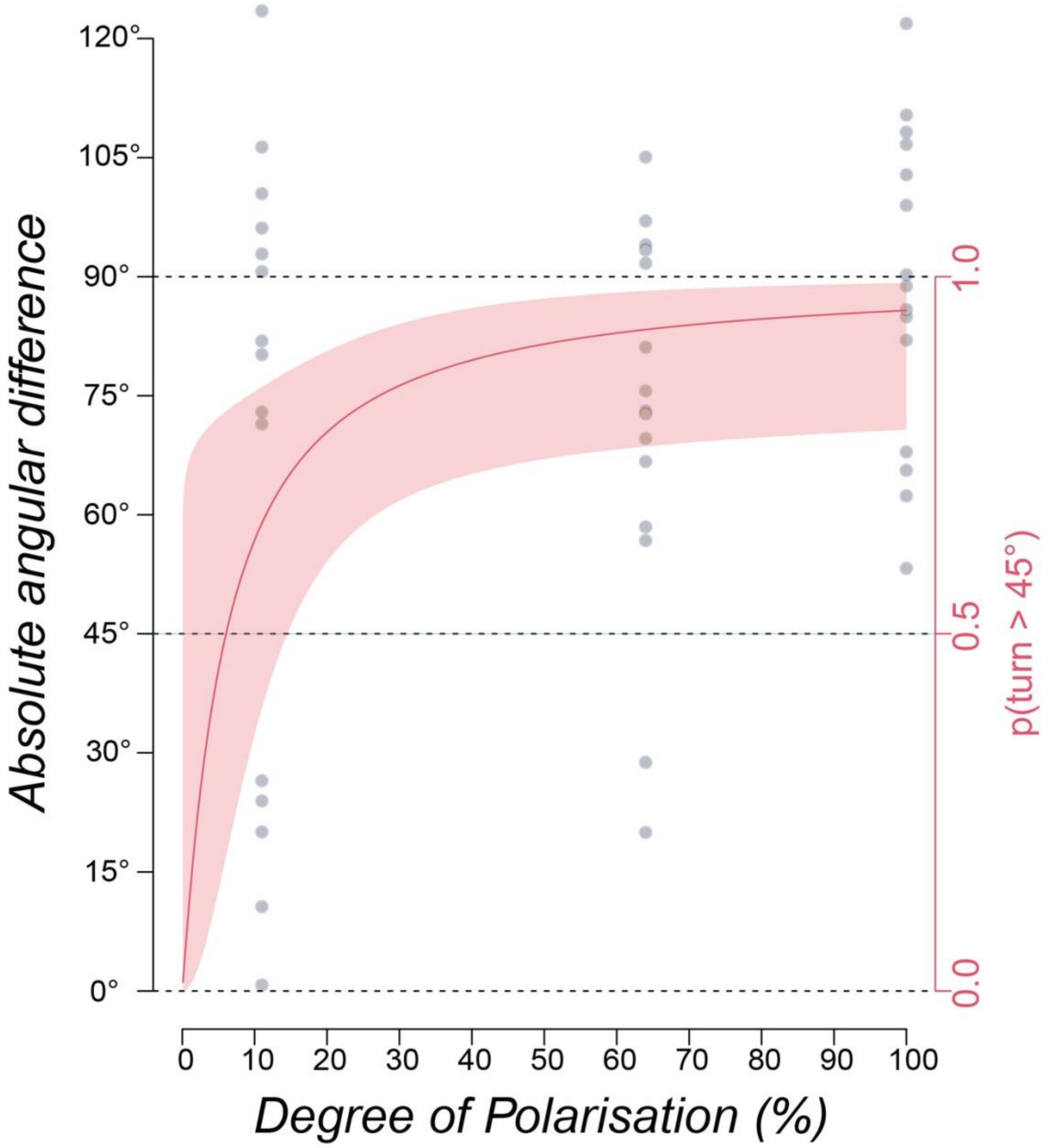

Fig. S1. A logistic regression fitted to the probability of a turn larger than 45° when the stimulus was turned by 90°. There was a significant increase in the probability of a turn with increasing degree of polarization (*11% polarisation*: 10/15 individuals; *64% polarisation*: 13/15 individuals; *100% polarisation*: 15/15). We modelled this relationship as a linear increase in the log-odds of a turn with the base 10 logarithm of degree of polarization in percent (following Foster et al., 2019). The fitted model is shown as a red line with red shaded 95% confidence intervals, superimposed on the original turn angles.

Journal of Experimental Biology • Supplementary information

Table S1. Summary of light measurements and behavioural outcome in each paradigm presented to the beetle. The table below gives a brief overview of the irradiance and wavelength measurements of each stimulus presented to the beetle, the behavioural response by the beetle in each paradigm, and the respective figure representing the result in the paper.

Stimulus setup

Stimulus	*Wavelength (nm)*	*Irradiance (photons cm^{-2} s^{-1})*
Ersatz sun	520	1.72×10^{13}
Dim ersatz sun	520	1.02×10^{12}
Overhead polarised light		
Cyan	505	2.39×10^{14}
UV	365	1.04×10^{15}
Dim overhead polarised light		
Cyan	505	2.39×10^{14}
UV	365	3.18×10^{13}

Response to stimulus

Condition	*Response to 90° positional change of stimuli?*	*Figure*
Ersatz sun	Yes	2A
Overhead light 11% polarised	Yes	2B
Overhead light 64% polarised	Yes	2C
Overhead light 100% polarised	Yes	2D
Ersatz sun + Overhead light 11% polarised	Yes	4A
Ersatz sun + Overhead light 64% polarised	Cannot be determined	4B
Ersatz sun + Overhead light 100% polarised	No	4C
Ersatz sun + Dim overhead light 64% polarised	Yes	4D

Journal of Experimental Biology • Supplementary information

Dim ersatz sun + Overhead light 64% polarised	No	4E
Dim overhead light 64% polarised	Yes	4F
Dim ersatz sun (no overhead light)	Yes	4G
Dim ersatz sun	No	4H

Journal of Experimental Biology • Supplementary information
